# Multimodal epigenetic sequencing analysis (MESA) of cell-free DNA for non-invasive colorectal cancer detection

**DOI:** 10.1186/s13073-023-01280-6

**Published:** 2024-01-16

**Authors:** Yumei Li, Jianfeng Xu, Chaorong Chen, Zhenhai Lu, Desen Wan, Diange Li, Jason S. Li, Allison J. Sorg, Curt C. Roberts, Shivani Mahajan, Maxime A. Gallant, Itai Pinkoviezky, Ya Cui, David J. Taggart, Wei Li

**Affiliations:** 1 Division of Computational Biomedicine, Department of Biological Chemistry, School of Medicine, University of California, Irvine, CA 92697 USA; 2 School of Biology and Basic Medical Sciences, Soochow University, Suzhou, 215123 P. R. China; 3 Helio Genomics, Inc, Irvine, CA 92618 USA; 4 State Key Laboratory of Oncology in South China, Guangdong Provincial Clinical Research Center for Cancer, Sun Yat-sen University Cancer Center, Guangzhou, 510060 P. R. China; 5 Guangzhou Youze Biological Pharmaceutical Technology Company Ltd, Guangzhou, 510005 P. R. China

**Keywords:** Liquid biopsy, Cancer detection, DNA methylation, Nucleosome, Polyadenylation

## Abstract

**Background:**

Detecting human cancers through cell-free DNA (cfDNA) in blood is a sensitive and non-invasive option. However, capturing multiple forms of epigenetic information remains a technical and financial challenge.

**Methods:**

To address this, we developed multimodal epigenetic sequencing analysis (MESA), a flexible and sensitive approach to capturing and integrating a diverse range of epigenetic features in cfDNA using a single experimental assay, i.e., non-disruptive bisulfite-free methylation sequencing, such as Enzymatic Methyl-seq. MESA enables simultaneous inference of four epigenetic modalities: cfDNA methylation, nucleosome occupancy, nucleosome fuzziness, and windowed protection score for regions surrounding gene promoters and polyadenylation sites.

**Results:**

When applied to 690 cfDNA samples from 3 colorectal cancer clinical cohorts, MESA’s novel modalities, which include nucleosome fuzziness, and genomic features, including polyadenylation sites, improve cancer detection beyond the traditional epigenetic markers of promoter DNA methylation.

**Conclusions:**

Together, MESA stands as a major advancement in the field by utilizing comprehensive and complementary epigenetic profiles of cfDNA for effective non-invasive cancer detection.

**Supplementary Information:**

The online version contains supplementary material available at 10.1186/s13073-023-01280-6.

## Background

Cancer has long been a leading cause of death worldwide. While research on cancer treatment continues to make progress in reducing cancer mortality, early detection provides the best opportunity to improve patient survival and lower treatment cost [1]. Recently, the analysis of circulating cfDNA — degraded DNA fragments in blood plasma originating primarily from the apoptosis of normal and diseased cells — has shown great potential for early cancer detection [2–4]. Using these liquid biopsies (non-invasive blood cfDNA-based detection methods) in routine screening is central to increasing surveillance adherence, identifying cancers in early curable stages, and ultimately reducing worldwide cancer mortality. One such approach is the whole-genome sequencing of cfDNA, which provides genetic information, such as somatic mutations and copy number variations [5, 6]. However, detecting cancer-specific genetic alterations is challenging due to the limited number of detectable changes and low fraction of circulating tumor DNA in patient blood samples [2, 5, 7, 8].

Aside from genetic alterations, cfDNA methylation has been shown as a promising biomarker for early cancer detection, as aberrant DNA methylation has been frequently reported in cancer cells and may occur early in tumorigenesis [9–12]. Recent studies showed that methylation has the best performance among those evaluated [13, 14] for cancer detection by performing simultaneous analysis of genetic alterations and methylations in cfDNA. Currently, the gold standard for the detection of DNA methylation is bisulfite sequencing. However, this harsh bisulfite treatment degrades a significant fraction of the DNA, resulting in biased genome coverage and increased sequencing cost [15]. Recently, the development of bisulfite-free DNA methylation sequencing methods, such as *E*nzymatic *M*ethyl-seq (EM-seq) and *T*ET-*a*ssisted *p*yridine borane *s*equencing (TAPS), have improved methylation sequencing quality and reduced sequencing cost [16–18]. Several studies compared bisulfite sequencing and EM-seq from the same cfDNA samples and found that methylation levels were similar between the two methods. However, EM-seq outperformed bisulfite sequencing in various metrics such as DNA damage, conversion efficiency, alignment quality, coverage, and sensitivity [19–21]. Furthermore, EM-seq was effective with lower input DNA and could preserve fragmentation patterns, making it a suitable method for evaluating the cfDNA methylome in both research and clinical settings.

Circulating cfDNA primarily consists of nucleosome-associated fragments that largely retain the chromatin structure information of the cells from which they originate [22, 23]. As cfDNA is degraded by endonucleases before being released into the bloodstream, closed chromatin regions with dense nucleosomes are particularly well-protected against enzymatic degradation, while open chromatin regions are more sensitive to endonuclease activity [22]. Several studies have developed methods utilizing chromatin-associated features for the non-invasive detection or monitoring of cancers, including nucleosome occupancy [24, 25], *w*indow *p*rotection *s*core (WPS) [22], and fragmentation profile [23, 26]. However, these methods rely on whole genome sequencing and thus do not provide further epigenetic information.

**Fig. 1:**
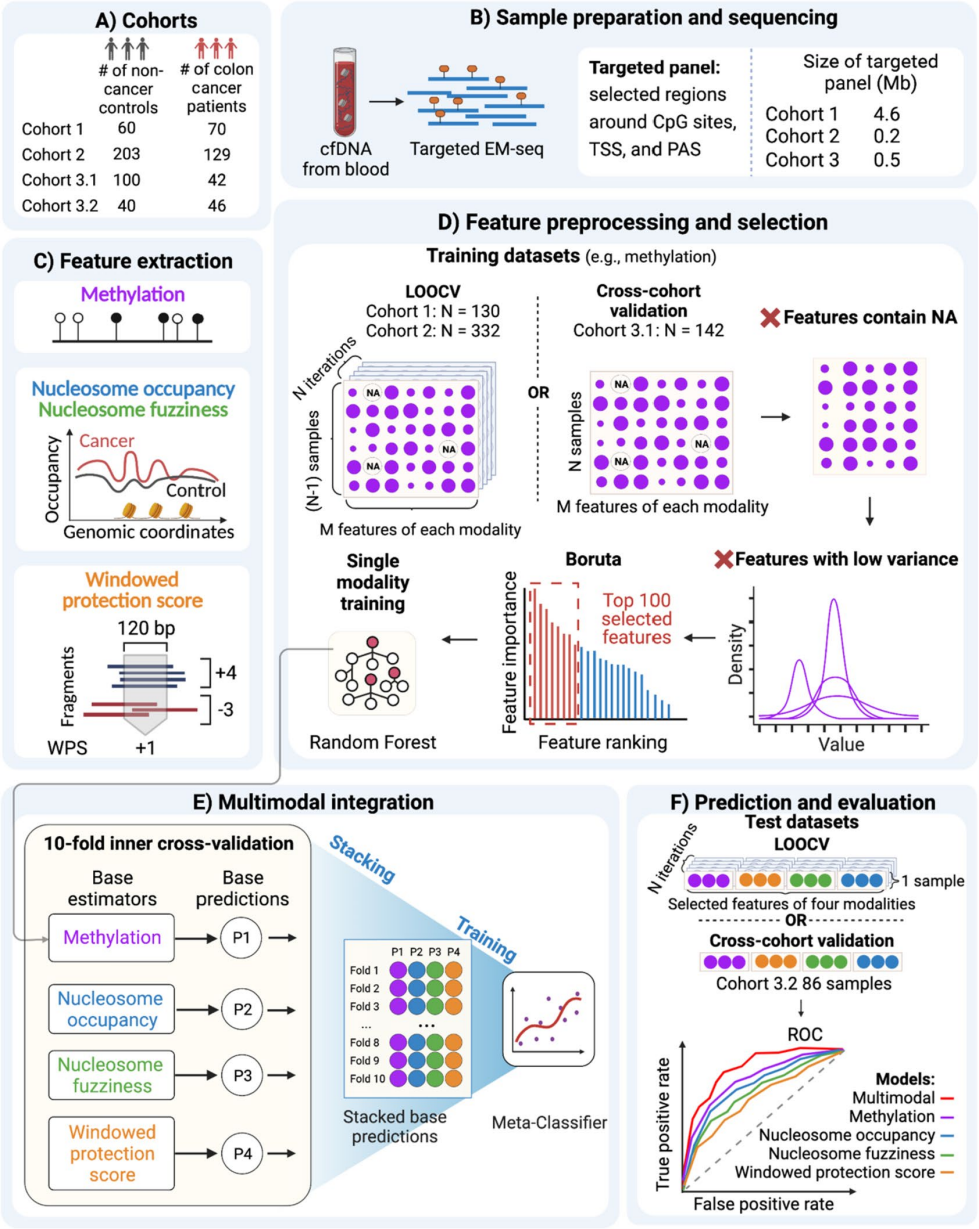
Schematic diagram displaying the design of MESA. cfDNA is isolated from blood samples of three cohorts (cohort 3 was split into cohort 3.1 and cohort 3.2 for cross-cohort validation) and then processed to generate targeted EM-seq libraries using three targeted panels. Analysis of the EM-seq data enables the extraction of four modalities: cfDNA methylation (purple), nucleosome occupancy (blue), nucleosome fuzziness (green), and windowed protection score (orange). Then, the feature processing and selection are performed for each modality separately. Firstly, features that contain NA or have low variance are removed. Next, the Boruta algorithm is used for feature ranking, and the top-ranking features (shown in red) are selected for the following analysis. Selected features are used for tenfold inner cross-validation for each modality to get the base predictions. Finally, by stacking and training the base predictions, we get a multimodal machine learning model which outperforms the single-modality models in cancer detection

Recently, the non-destructive nature of EM-seq and TAPS enabled the combination of two epigenetic modalities based on low-coverage whole-genome methylation sequencing (Additional file 1: Table S1). In particular, cfDNA TAPS [27] provided DNA methylation and fragmentation for 85 samples from cancer patients, cirrhosis patients, pancreatitis patients, and healthy controls. Similarly, EM-seq-based cfDNA sequencing [21] measured DNA methylation and nucleosome occupancy for 12 samples from chronic kidney disease patients and healthy controls. Despite this progress, these two methods are largely limited by small sample sizes and fail to utilize the full spectrum of epigenetic information from cfDNA. Here, we introduce a multimodal epigenetic sequencing analysis (MESA) of cfDNA (Fig. 1) for 690 colorectal cancer and control samples from three cohorts with deep targeted EM-seq. MESA can simultaneously infer four highly complementary epigenetic modalities, namely (1) cfDNA methylation, (2) nucleosome occupancy, (3) nucleosome fuzziness, and (4) WPS across gene promoters and polyadenylation sites. The introduction of novel modalities (e.g., nucleosome fuzziness) and genomic features (e.g., polyadenylation sites) in MESA significantly improved cancer detection beyond traditional epigenetic markers (e.g., promoter DNA methylation).

## Methods

### Study cohort

There are three clinical cohorts in this study, namely cohort 1, cohort 2, and cohort 3 (Additional file 1: Tables S2-S4). Cohort 1 comprised 70 patients diagnosed with colorectal cancer and 60 control individuals without colorectal cancer. Cohort 1 subjects were recruited at clinical sites within the USA through the ELITE Study (NCT05181826) or were obtained through the following contract research organizations: BioIVT (Westbury, NY, USA), BioOptions (Brea, CA, USA), Discovery Life Sciences (Boston, MA, USA), and DX Biosamples (San Diego, CA, USA). Cohort 2 comprised 129 patients diagnosed with colorectal cancer and 203 control individuals without colorectal cancer. Cohort 2 subjects were enrolled at the Sun Yat-sen University Cancer Center (Guangzhou, China). Cohort 3 comprised 88 patients diagnosed with colorectal cancer and 140 control individuals without colorectal cancer (there were 53 overlapped with subjects of cohort 1). Cohort 3 has been divided into two sub-cohorts: cohort 3.1, which comprises newly recruited subjects, and cohort 3.2, which includes the original subjects from cohort 1. Due to a shortage of cfDNA material in cohort 1, we could only obtain 7 non-cancer control samples in cohort 3.2. To carry out the cross-cohort validation analysis, we added 33 additional control samples with matching age and gender to the original control samples in cohort 1, to be included in cohort 3.2. As a result, the final composition of cohort 3.1 includes 42 cancer patients and 100 control individuals, while cohort 3.2 consists of 46 cancer patients and 40 control individuals. Cohort 3 subjects were recruited at the same sites as cohort 1. We did a principal component analysis based on DNA methylation data for cohort 1 and cohort 3 and noticed that the variation caused by different collection sites was insignificant (Additional file 2: Fig. S1). Subjects diagnosed with colorectal cancer were diagnosed according to current clinical practices. We required that the control subjects had no clinical history or symptoms of colorectal cancer and excluded the possibilities of colorectal cancers and precancerous lesions using colonoscopy. Cohort 1 and cohort 2 were used for leave-one-out cross-validation analysis separately. Cohort 3 was used for cross-cohort analysis. All specimen collection protocols were approved by the respective Institutional Review Board (IRB). For all three cohorts, informed consent was obtained from all patients following the Declaration of Helsinki Ethical Principles for medical research involving human subjects.

### Collection and preparation of samples

Cohort 1 and cohort 3 specimens were drawn into PAXgene cfDNA tubes (PreAnalytiX) and shipped to a central Helio Genomics laboratory (USA) using custom specimen collection and shipping kits (Helio Genomics, USA). The whole blood specimens were then processed to cleared plasma by centrifugation and stored at approximately – 80 °C until analysis. Cohort 2 specimens were drawn into KANGJIAN blood collection tubes at the corresponding hospital. Samples were shipped to a Youze labortory (Guangzhou Youze Biological Pharmaceutical Technology Company Ltd., China) with dry ice and stored at approximately – 80 °C until analysis.

### Targeted sequencing panel design

TCGA-COAD and TCGA-READ 450 K methylation array data were downloaded from the UCSC Xena database (https://tcga.xenahubs.net) [28]. Additional DNA methylation array datasets were downloaded from GEO with accession numbers GSE53051 [29], GSE48684 [30], and GSE42752 [31] (https://www.ncbi.nlm.nih.gov/geo/). All datasets were processed by a custom script to identify CpG sites with significant methylation differences between cancerous and adjacent normal tissues. A total of 9599 significantly differentially methylated CpG sites in the colorectal cancer samples, along with 200 markers mentioned in the literature, were selected. A list of 150 bp genomic regions centered on each of the selected CpG sites was designed for targeted sequencing. Additionally, 912 promoter regions (Transcription start site ± 1 kb) and 365 polyadenylation regions (polyadenylation site ± 1 kb) of the curated cancer-related genes were added to the targeted panel. With the repeat elements and ENCODE blacklist regions removed [32], the size of the version 1 colorectal cancer targeted panel (used on cohort 1) was about 4.6 Mb (Additional file 1: Tables S5, S6). To design the panel in cohort 2, we selected the top-performing 1000 methylation markers from the initial set in cohort 1. Furthermore, 355 top nucleosome features in cohort 1 were selected based on a prediction AUC of >  = 0.725. The shrinking version 2 colorectal cancer targeted panel (used on cohort 2) was about 220 kb (Additional file 1: Tables S7, S8). To design the panel in cohort 3, top methylation features and nucleosome organization features were selected based on F-statistics in each fold of the repeated fivefold cross validation from the initial set in cohort 1. The most frequently selected features were finally included. Additionally, promoter regions of colorectal tissue specific genes and differentially expressed genes in cancer patients identified from the TCGA dataset were also included in the panel. The shrinking version 3 colorectal cancer targeted panel (used on cohort 3) was about 472 kb (Additional file 1: Tables S9, S10). All targeted panels were synthesized by Twist Bioscience (USA).

### Targeted EM-seq of cfDNA

The Helio ECLIPSE™ platform was used to analyze cfDNA extracted from patient specimens as previously described [33]. Briefly, total cfDNA was isolated from specimens by using either (cohort 1 and cohort 3) a QIAsymphony DSP Circulating DNA Kit (QIAGEN, USA) or (cohort 2) the EliteHealth cfDNA Extraction Kit (EliteHealth, China). Spike-in control unmethylated Lambda DNA was sheared down into about 170 bp by sonication. A total of 5 ng cfDNA along with 0.2 pg of unmethylated Lambda DNA per specimen was used to prepare the barcoded NGS libraries using the NEB Next Enzymatic Methyl-seq Kit (New England Biolabs, USA) according to the manufacturer’s instructions. The libraries were then hybridized with a custom set of capture probes (Twist Bioscience, USA) to capture the targeted library sequences using the Twist Fast Hybridization and Wash Kit, along with the Twist Universal Blocker. Then, a PCR step with 12 cycles was applied to the targeted library sequences for library amplification. The PCR product was purified and quantified by Thermo Fisher Qubit 4 Fluorometer. Only products that had a volume higher than 30 μL and concentration high than 2 ng/μL were kept for the following steps. Finally, the distribution of the library fragment length was measured by Agilent 4200 TapeStation. Only high-quality libraries with peak size between 300 and 350 bp (corresponding to 130–180 bp cfDNA fragments and 170 bp adapter) were kept for sequencing. The captured libraries were then supplemented with a 20% PhiX genomic DNA library to increase base calling diversity and submitted for sequencing using Illumina NovaSeq 6000 instruments as 2 × 150 bp reads.

### Targeted EM-seq data processing and quality control

Raw sequencing reads were first trimmed by TrimGalore (https://github.com/FelixKrueger/TrimGalore, –paired -q 20 –clip_R1 5 –clip_R2 10 –three_prime_clip_R1 30 –three_prime_clip_R2 30; v0.6.5) to remove low-quality reads and potential adaptor contamination. Then, the remaining reads were aligned to the hg19 human genome reference using BSMAP (v2.90) [34]. The aligned reads were further processed by Samtools (v0.1.19) [35] and deepTools (v3.5.0) [36] to only keep primarily mapped reads with fragment sizes between 80 and 200 bp to remove potential genomic DNA contamination from normal blood cells. This final file served as the input file for all the following processes except fragment size distribution analysis, which used reads without a size filter. Spike-in unmethylated lambda DNA was used to control for C to T conversion efficiency. Samples with lambda methylation levels of more than 1% (CT conversion rate less than 99%) were removed from the downstream analysis. In total, there were 5 samples removed because of low CT conversion rate including 2 from cohort 1 and 3 from cohort 2.

### Multimodal feature extraction from targeted EM-seq of cfDNA

We extracted four types of features: cfDNA methylation, nucleosome occupancy, nucleosome fuzziness, and WPS.

- cfDNA methylation: Conventional methylation ratio was calculated by Methratio.py (BSMAP, v2.90) [34] from aligned bam files for the target CpG sites.
- Nucleosome occupancy: Occupancy values were calculated using DANPOS2 (v2.2.2) [37]. For cohort 2 and cohort 3, the average value for each nucleosome organization target region was calculated using bigWigAverageOverBed from UCSC tools (v393) [38]. Due to the relatively long target regions of cohort 1 (2 kb), we split each target region into 1 kb sliding windows with 10 bp steps. Then, for each sliding window, we calculated the average nucleosome occupancy.
- Nucleosome fuzziness: Fuzziness values were calculated using DANPOS2. For each nucleosome organization target region (1 kb sliding windows for cohort 1), we calculated the average fuzziness of all the nucleosomes whose center is located within the region.
- WPS: Average WPS was calculated for each targeted region as described previously [22].

### Single modality machine learning models for cancer detection

We trained machine learning models for cohort 1 and cohort 2 using the same procedure. All the models were trained and evaluated using the leave-one-out cross-validation method. Briefly, all the *N* samples were divided into training and test samples for *N* iterations, where the number of test samples = 1 and the number of training samples = *N* -1. Since missing values could reduce the accuracy of the machine learning model, we excluded features with missing values in each iteration. Next, low-variance features were removed before training the model in the remaining training datasets. The feature’s predictive capabilities and the model's performance were then assessed using the test datasets. Finally, the results of all the *N* iterations were aggregated together to calculate performance metrics. The Random Forest classifier from scikit-learn package (v0.24.2) [39] was used for the single modality model construction.

### Feature selection

For each of the four modalities, we used the Boruta algorithm in the BorutaPy package (v0.3) [40] in each iteration of leave-one-out cross-validation to determine feature importance. Specifically, we created copies of the original features and randomly permuted their values. Then, we trained a machine learning model using Random Forest on the datasets with both original and permuted features. We evaluate the importance of each real feature and mark it as “confirmed important” when it showed significantly higher importance than its permuted version. If a feature was less important than its permuted version, it was considered “unimportant” and was eliminated from further consideration. We repeated the steps above until all features were either confirmed important or unimportant. Finally, the Boruta algorithm ranked the features based on their importance, with the “confirmed important” features having the highest importance. Finally, we selected the top 100 features for each modality for model training and prediction.

### Multimodal machine learning model for cancer detection

We built the multimodal machine learning model using the model-based multimodal integration strategy [41]. For each of the four modalities, we selected a feature subset using the Boruta algorithm and trained a Random Forest classifier using the complete training dataset as a base estimator. Specifically, in each iteration of leave-one-out cross-validation, we used the tenfold cross-validation (CV) inside the training dataset, and we stacked all base predictive probabilities in each CV of four modalities as input to a meta-classifier to make prediction on the corresponding leave-one-out cross-validation test dataset. This ensemble learning approach could preserve unique information from different modalities and provide complementary information across different types of features.

### cfDNA TAPS data processing and machine learning models for cancer detection

The cfDNA TAPS data was processed in the same manner as the original paper [27]. Raw sequenced reads were trimmed using TrimGalore (v0.6.5, https://github.com/FelixKrueger/TrimGalore) to remove adapter and low-quality bases. Trimmed reads were aligned to the hg19 human reference genome using bwa mem (v0.7.17) [42]. The alignment files were filtered to remove low mapping quality (MAPQ < 20) as well as duplicate reads using alignmentSieve from deepTools (v3.5.0) [36]. MethylDackel extract (v0.6.1, https://github.com/dpryan79/MethylDackel) was used for methylation calling. CpG sites that overlapped common single-nucleotide polymorphism (SNP) [43] (https://ftp.ncbi.nih.gov/snp/organisms/human_9606_b151_GRCh37p13), blacklisted regions [32], centromeres, and sex chromosomes were excluded from downstream analysis.

Next, we extracted three types of features: DNA methylation, nucleosome occupancy, and WPS. (1) DNA methylation: The methylation ratio was calculated using the number of methylated CpGs divided by the total number of sequenced CpGs for each promoter and enhancer region. The promoter and enhancer regions were downloaded from Ensemble [44] (http://ftp.ensembl.org/pub/grch37/release-100/regulation/homo_sapiens/homo_sapiens.GRCh37.Regulatory_Build.regulatory_features.20191101.gff.gz). (2) Nucleosome occupancy: Occupancy values were calculated using DANPOS2. Average values of the 1 kb regions surrounding TSSs and polyadenylation sites of all RefSeq annotated genes [45] were calculated. The locations of polyadenylation sites were downloaded from PolyA_DB (version 3) [46]. Due to the relatively low coverage of cfDNA TAPS data, we removed features that had occupancy values lower than the mean of all values in at least one sample. (3) WPS: Average WPS was calculated for the 1 kb regions surrounding TSSs and polyadenylation of all RefSeq annotated genes, which were also used for nucleosome occupancy.

We then trained both two-class (distinguishing cancer (HCC or PDAC) and control samples) and three-class models (distinguishing HCC, PDAC, and control samples) using the same procedure as for targeted EM-seq data. For the three-class models, we used accuracy instead of AUC as the performance metric.

### Cross-cohort validation analysis

We performed cross-cohort validation on cohort 3. Briefly, after feature extraction for both cohorts, we performed feature preprocessing, selection, and multimodal integration as described before. Then, we trained the model on cohort 3.1 and calculated the predictive performance with the trained model on cohort 3.2.

### SMAC-seq data processing

SMAC-seq data for the human GM112878 cell line was downloaded from https://zoharshiponh.s3.amazonaws.com/NMETH_2020/index.html. Then, the data was processed by following the steps in the original paper [47] using scripts from https://github.com/georgimarinov/SMAC-seq-scripts. Next, nucleosome occupancy could be calculated based on the ratio of methylated A/unmethylated A. The nucleosome occupancy profile from SMAC-seq around all polyadenylation sites downloaded from PolyA_DB (version 3) [46] was visualized using R (v4.0.4).

## Results

### MESA cohorts

To systematically demonstrate the performance of MESA, we designed three targeted EM-seq panels of different scales for three clinical cohorts, namely cohort 1 (*n* = 130), cohort 2 (*n* = 332), and cohort 3 (*n* = 228) (Fig. 1, Additional file 1: Tables S2-S4). We used cohorts 1 and 2 individually to showcase the versatility and robustness of the MESA method and used cohort 3 for cross-cohort analysis with cohort 1. The target regions included a custom-designed methylation panel and a nucleosome organization panel with 1-kb regions surrounding both transcription start sites (TSSs) and polyadenylation sites (PASs) of cancer-related genes (Methods; Additional file 1: Tables S5-S10). The methylation panel included significantly differentially methylated CpG sites from the TCGA 450 K colorectal cancer cohort and CpG markers collected from the literature. Novel to our panel design is the introduction of polyadenylation sites, whose alternative regulation is frequently reported to be involved in tumorigenesis [48–51]. Since nucleosome occupancy around polyadenylation sites is also associated with alternative polyadenylation regulation [52–54], we predicted that its inclusion would contribute to the improvement of the cancer detection model’s performance. While our target panel was specifically designed for colorectal cancer, its design strategies allow for easy adaption to other cancer types or non-cancer diseases. In contrast to low-pass whole-genome methylation sequencing such as cfDNA TAPS [27] (mean coverage of 11.6 ×), this targeted design allowed us to perform deeper sequencing with a mean coverage of 74.2 × (range from 41 to 123 ×) for cohort 1, a mean coverage of 200.3 × (range from 76 to 570 ×) for cohort 2, and a mean coverage of 157.4 × (range from 78 to 314 ×) for cohort 3 at a relatively low cost. Next, we assessed the quality of the sequencing data based on non-human internal spike-in controls with known unmethylated CpG sites (CpG-unmethylated lambda DNA). Only samples with a conversion efficiency of at least 99% were kept for analysis, corresponding to less than 1% methylation detected in the unmethylated lambda DNA.

### cfDNA methylation in MESA enables accurate detection of colorectal cancer

**Fig. 2:**
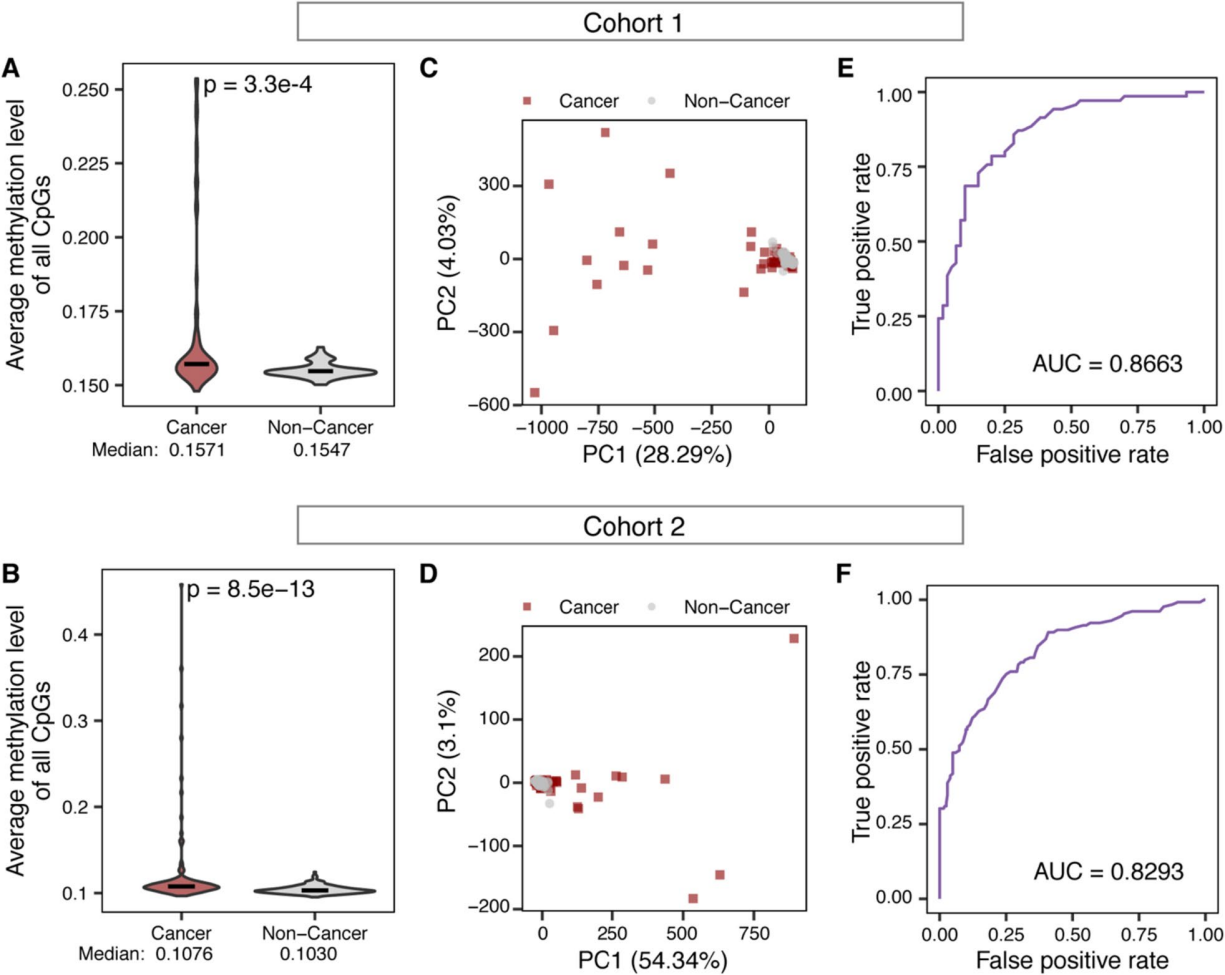
Differential cfDNA methylation between cancer and non-cancer samples enables accurate cancer detection. **A-B** The average methylation level of all target CpG sites in cancer patients (Cancer) and controls (Non-Cancer) from cohort 1 (**A**) and cohort 2 (**B**). **C-D** Scatter plots showing PC1 and PC2 from PCA of methylation level of all target CpG sites in cancer patients (Cancer) and controls (Non-Cancer) of cohort 1 (**C**) and cohort 2 (**D**). The percentage of variances explained by each PC is shown in the parentheses. **E-F** Receiver operating characteristic (ROC) curves of model performance based on the methylation level of CpG sites for cohort 1 (**E**) and cohort 2 (**F**). The results from 130 iterations for cohort 1 and 332 iterations for cohort 2 of leave-one-out cross-validation analysis were shown

As a baseline, we first explored the effectiveness of cfDNA methylation features alone in distinguishing between cancer patients and non-cancer controls. We observed that the average methylation level of all target CpG sites was elevated in cancer samples compared to non-cancer controls (Fig. 2A and B). This observation is consistent with the fact that the targeted CpG sites are primarily located in promoter regions, which are known to be frequently hypermethylated in cancers [55]. Principal component analysis (PCA) for cfDNA methylation levels in all target CpG sites showed reasonable separation in PC1 and PC2 (Fig. 2C and D). Next, we investigated the performance of these methylation features for colorectal cancer prediction using machine learning methods with leave-one-out cross-validation (Methods). Methylation alone achieved an impressive prediction of colorectal cancer in both cohorts based on random forest models (Fig. 2E and F, AUC (area under the curve) = 0.8663 for cohort 1 and AUC = 0.8293 for cohort 2). These results indicated that cfDNA methylation in MESA can be used to detect colorectal cancer with reasonable accuracy.

### MESA successfully captures nucleosome organization information

**Fig. 3:**
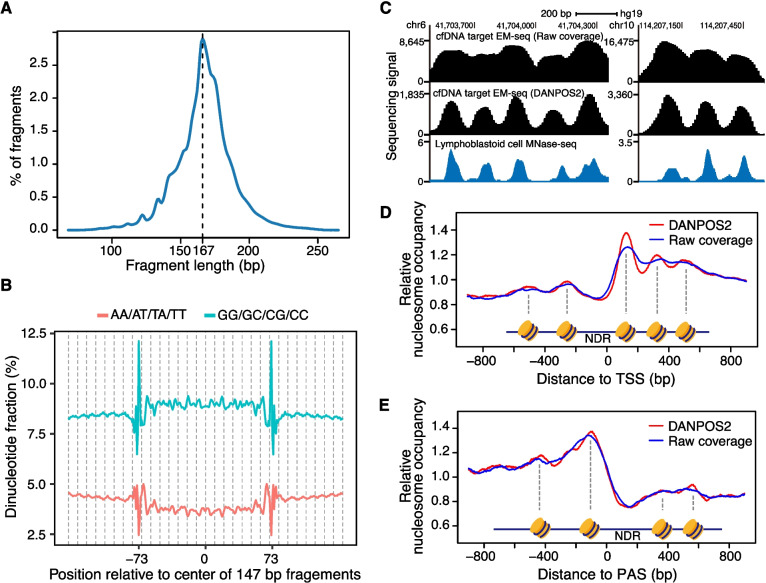
Nucleosome organization information from targeted EM-seq of cfDNA. **A** Fragment length distribution of sequenced cfDNA fragments. A peak value at 167 bp (black dashed line) is consistent with the association with nucleosomes. **B** The distribution of dinucleotide fraction across 147 bp fragments and the flanking genomic regions. **C** Genome browser tracks showing sequencing signals of targeted EM-seq of healthy cfDNA (cfDNA targeted EM-seq) and nucleosome calls generated by ENCODE project with accession number ENCSR000CXP (Lymphoblastoid cell MNase-seq). DANPOS2, occupancy values reported by DANPOS2. Raw coverage, occupancy values estimated by read coverage. **D-E** Aggregate lines showing nucleosome occupancy profiles across TSSs (**D**) and polyadenylation sites (**E**) of target genes. PAS, polyadenylation sites. NDR, nucleosome depleted regions. Relative nucleosome occupancy represents nucleosome occupancy normalized by the average value of the plotted regions. Results in this figure are based on merged targeted EM-seq data of 60 healthy controls from cohort 1

EM-seq preserves the integrity of cfDNA as compared to bisulfite conversion, enabling us to capture additional epigenetic information. From all the sequenced fragments merged from non-cancer controls, we observed a peak around 166 bp (corresponding to the length of DNA associated with a nucleosome and a linker histone) in the cfDNA fragment length distribution (Fig. 3A for cohort 1, Additional file 2: Fig. S2A, B for cohort 2 and cohort 3), which is consistent with that from cfDNA whole-genome sequencing data [22, 24]. The size distribution of fragments between the two US-collected cohorts (cohort 1 and cohort 3) did not differ significantly, as determined by a Kolmogorov–Smirnov test (*P* = 0.5944). Further supporting the association between cfDNA and nucleosomes, the dinucleotide frequency of these fragments showed a ~ 10 bp periodicity (Fig. 3B for cohort 1, Additional file 2: Fig. S3A, B for cohort 2 and cohort 3), which recapitulates key features of nucleosome-associated fragments digested by micrococcal nuclease [56]. Next, to accurately measure nucleosome organization profiles from cfDNA, we used the quantification method DANPOS2 [37, 57], a tool widely used for processing micrococcal nuclease digestion with deep sequencing (a technique used for profiling nucleosome landscape) data [58]. The occupancy profiles reported by DANPOS2 were concordant with nucleosome profiles from lymphoblastoid cells (Fig. 3C), indicating the targeted EM-seq successfully captured nucleosome information. Moreover, profiles reported by DANPOS2 had lower background noise compared with raw read coverage measurements, as shown by example regions (Fig. 3C) and the typical well-positioned nucleosomes around TSSs (Fig. 3D). Interestingly, we also observed a nucleosome-depleted region around polyadenylation sites and well-positioned nucleosomes flanking this region (Fig. 3E). To exclude potential AT bias of coverage-based sequencing data, we further used SMAC-seq [47], an enzymatic footprint strategy based on the ratio of methylated A and unmethylated A, to measure nucleosome occupancy. SMAC-seq data for the human GM112878 cell line also showed a nucleosome-depleted region around polyadenylation sites, which was even clearer than those from the coverage-based approaches (Additional file 2: Fig. S4). These results demonstrate that MESA successfully captures nucleosome organization information in both TSSs and polyadenylation sites.

### Nucleosome occupancy and fuzziness in MESA enable accurate detection of colorectal cancer

**Fig. 4:**
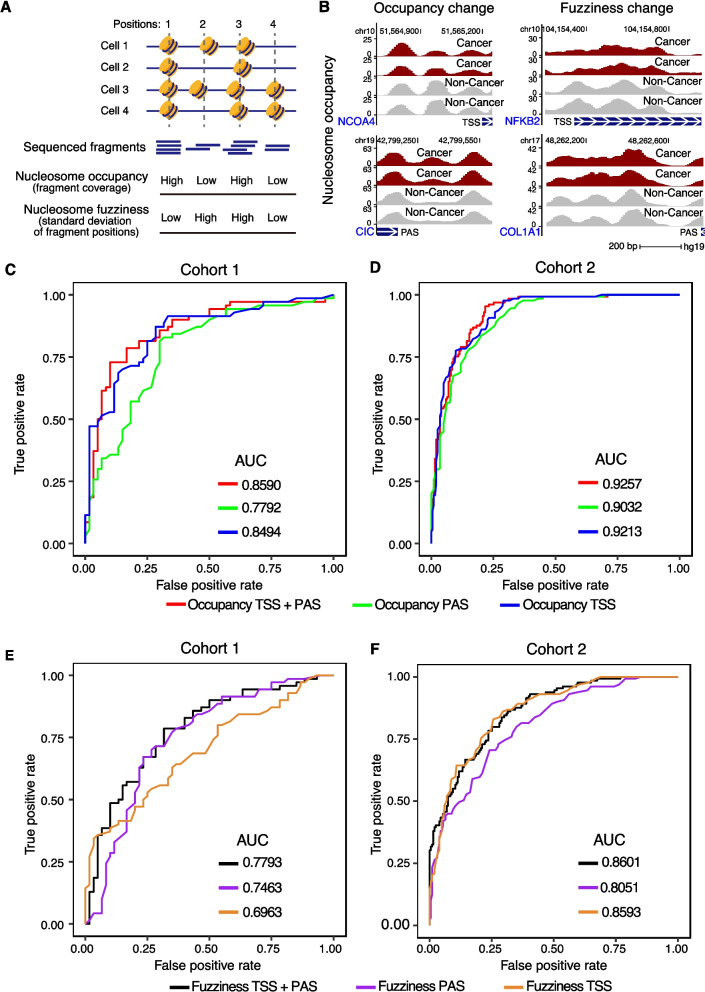
Accurate detection of cancer based on nucleosome occupancy and fuzziness. **A** A schematic diagram showing the differences between nucleosome occupancy and fuzziness for four example positions in four cells. **B** Genomic regions showing nucleosome occupancy (left panel) and fuzziness changes (right panel) between cancer and non-cancer samples. The top panel shows genome browser tracks of TSS target regions, and the bottom panel shows PAS target regions. For each panel, two example cancer and non-cancer samples are displayed. The blue boxes show the gene bodies with white arrows indicating the transcription directions. **C**, **D** ROC curves showing the model performances based on the nucleosome occupancy of TSS target regions (Occupancy TSS), PAS target regions (Occupancy PAS), or combination of the two (Occupancy TSS + PAS). **E**, **F** ROC curves showing the model performances based on nucleosome fuzziness of TSS target regions (Fuzziness TSS), PAS target regions (Fuzziness PAS), or combination of the two (Fuzziness TSS + PAS). PAS, polyadenylation site. For the ROC curves, results from 130 iterations for cohort 1 and 332 iterations for cohort 2 of leave-one-out cross-validation analysis were shown

Based on our findings that DANPOS2 could accurately measure nucleosome organization features from targeted EM-seq, we then investigated whether these features could be used for cancer detection. We derived two types of features from nucleosome organization: (1) nucleosome occupancy, which reflects the frequency with which nucleosomes occupy a given DNA region in a cell population; (2) nucleosome fuzziness, which is defined as the deviation of nucleosome positions within a region in a cell population and could reflect cell heterogeneity at the chromatin level (Fig. 4A). Both features were defined for each nucleosome organization target region (TSS and polyadenylation sites target regions) by DANPOS2 (Methods). We hypothesized that nucleosome occupancy and fuzziness might capture non-overlapping changes between cancer and control samples. Genome browser track visualization of four regions showed examples of either occupancy or fuzziness changes between cancer and control samples in cohort 1 (Fig. 4B). Particularly, these changes were found in both TSS (Fig. 4B, top panels) and polyadenylation (Fig. 4B, bottom panels) regions, emphasizing the importance of introducing polyadenylation site target regions in the MESA panel design.

Using the leave-one-out cross-validation method, we then investigated the predictive potential of nucleosome occupancy and fuzziness. Consistent with previous work [24], our model based solely on nucleosome occupancy of TSS target regions achieved an AUC of 0.8494 for cohort 1 and 0.9213 for cohort 2 (Fig. 4C and D). Interestingly, adding polyadenylation site target regions further improved model performance, as demonstrated by the enhanced AUC after combining nucleosome occupancy features of TSS and polyadenylation site target regions (Fig. 4C and D; AUC = 0.8590 for cohort 1 and AUC = 0.9257 for cohort 2). To the best of our knowledge, this is the first time that nucleosome occupancy around polyadenylation regions from cfDNA has been utilized in cancer detection, as most of the previous studies utilizing nucleosome-associated features did not utilize polyadenylation sites and primarily focused on gene promoters (Additional file 1: Table S11). Another novelty of our design is the introduction of nucleosome fuzziness, which reflects cell heterogeneity at the chromatin level [37, 59]. Nucleosome fuzziness based on cfDNA may differentiate cancer from controls, as cancerous tissue is typically more heterogeneous than normal tissue [60, 61]. To exclude the possibility that nucleosome fuzziness had been previously reported under a different name, we examined two measurements derived from the nucleosome-associated metrics, namely WPS [22] and *o*rientation-aware *c*fDNA *f*ragmentation (OCF) [62]. However, our results demonstrate that nucleosome fuzziness is distinct from these metrics, as evidenced by its low per-sample correlations (absolute value less than 0.5) and differing predictive probabilities (Spearman correlation of 0.61 for nucleosome fuzziness and WPS, Spearman correlation of 0.51 for nucleosome fuzziness and OCF, as shown in Additional file 2: Fig. S5). Therefore, the inclusion of nucleosome fuzziness and polyadenylation sites remain the primary novel contributions in our study. Our model based solely on nucleosome fuzziness showed good performance in cancer detection, and the addition of polyadenylation sites further improved the model’s performance (Fig. 4E and F; AUC = 0.7793 for cohort 1 and AUC = 0.8601 for cohort 2). These results suggested that the new modality (nucleosome fuzziness) and genomic feature (polyadenylation sites) introduced in MESA are effective for cancer detection.

### Integrating multimodal epigenetic features in MESA enhances cancer detection

**Fig. 5:**
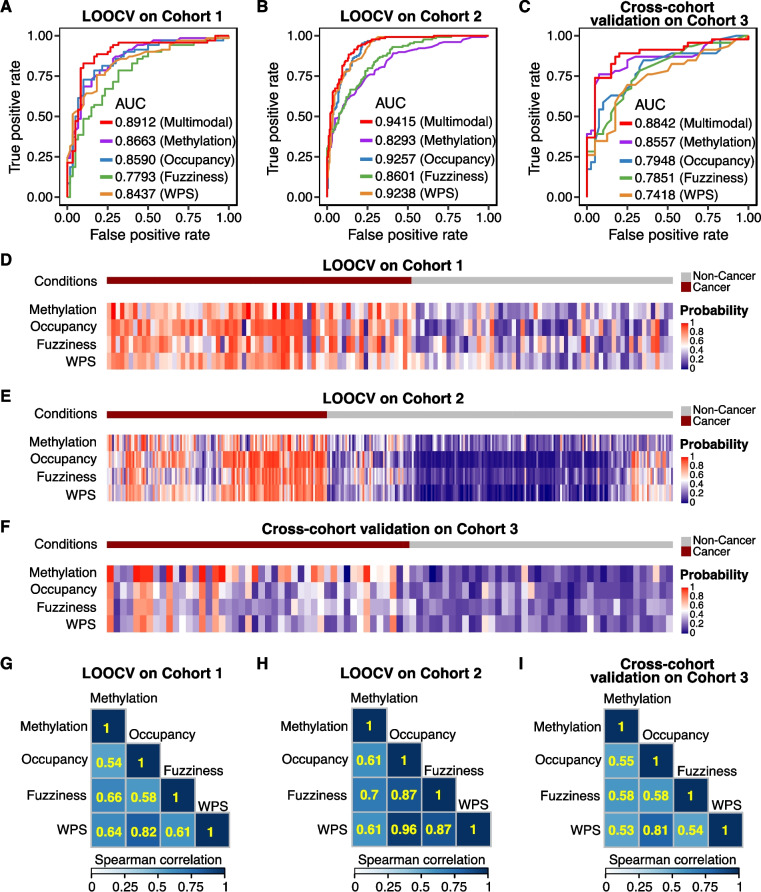
Multimodal epigenetic analysis from MESA improves the performance of cancer detection model. **A**–**C** ROC curves showing model performances based on different modalities. Methylation, methylation ratio of all target CpGs; Occupancy, nucleosome occupancy of all TSS and polyadenylation target regions; Fuzziness, nucleosome fuzziness of all TSS and polyadenylation target regions; WPS, average WPS for all target regions; Multimodal, the combination of all four types of features. **D**–**F** Heatmaps showing the predicted probabilities of single modality models for each sample. The probability represents the predicted probability of classifying the sample to the cancer group. **G**–**I** Heatmaps showing pairwise Spearman correlations of the predicted probability of all samples between different types of features. The Spearman correlation values are labeled on the heatmaps. LOOCV on cohort 1, the leave-one-out cross-validation analysis results from 130 iterations on cohort 1. LOOCV on cohort 2, the leave-one-out cross-validation analysis results from 332 iterations on cohort 2. Cross-cohort validation on cohort 3, results of models trained on cohort 3.1 and validated on cohort 3.2

We next investigated the integration of multimodal features captured by MESA for cancer detection. In addition to DNA methylation, nucleosome occupancy, and nucleosome fuzziness features we previously introduced, we also included WPS, which has been widely used for cancer detection [21, 22, 63, 64]. Using leave-one-out cross-validation, we found that the integrated models had the highest AUC, sensitivity (at 90% specificity), and F1 score compared to the four single modality models (Fig. 5A and B; Additional file 1: Tables S12, S13), highlighting the benefits of incorporating multimodal information in cancer prediction. When evaluating models based on the cancer stages, the multimodal model still outperformed single modality models (Additional file 1: Table S14). By visualizing the predicted probability of classifying each sample to the cancer group, we found a similar pattern for the four single modality models (Fig. 5D and E), suggesting that each modality concordantly predicted the same classification for most samples. Additionally, when examining the correlations between the probabilities of different single-modality models, we found correlations as low as 0.53 (Fig. 5G and H), indicating that single-modality models may capture complementary information for cancer detection. The observed improved performance of the integrated model is consistent with the fact that the integration of single modalities combines complementary information.

### MESA for cross-cohort analysis

To demonstrate the robustness of MESA across cohorts, we included a third cohort (cohort 3, *n* = 228) for cross-cohort analysis with cohort 1. To achieve a more cost-effective deep sequencing coverage, we reduced the targeted panel of cohort 3 based on the panel used in cohort 1. To minimize batch effects, we re-sequenced the samples of cohort 1 using the modified panel. As a result, cohort 3 has been divided into two sub-cohorts: cohort 3.1, which comprises newly recruited subjects (42 cancer patients and 100 control individuals), and cohort 3.2, which includes the original subjects from cohort 1 (46 cancer patients and 40 control individuals) (Methods). Our MESA method still demonstrated strong performance, as evidenced by the cross-cohort analysis results, where the model was trained on cohort 3.1 and validated on cohort 3.2 (Fig. 5C). Furthermore, each modality captured complementary information and predicted the same classification for most samples in the cross-cohort analysis (Fig. 5F and I).

### MESA for other bisulfite-free DNA methylation sequencing methods

**Fig. 6:**
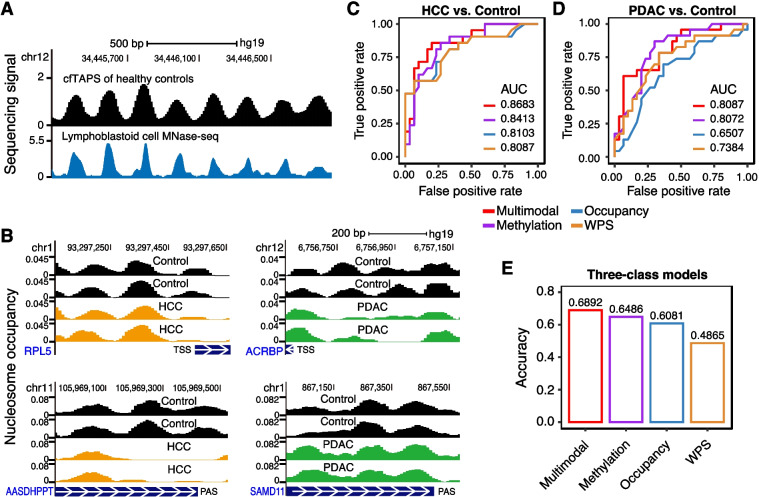
Multimodal epigenetic analysis of cfDNA TAPS improves the performance of cancer detection model. **A** Genome browser tracks showing sequencing signals of cfDNA TAPS of controls (cfTAPS of healthy controls) and nucleosome calls generated by ENCODE project with accession number ENCSR000CXP (Lymphoblastoid cell MNase-seq). Sequencing signals from cfDNA TAPS are calculated by DANPOS2. **B** Genomic regions showing nucleosome occupancy changes between HCC (left panel) or PDAC (right panel) and control samples. Nucleosome occupancy is calculated by DANPOS2. The top panel shows tracks of regions surrounding TSSs, and bottom panel shows regions surrounding polyadenylation sites. For each panel, two example cancer and control samples are displayed. The blue boxes show the gene bodies with white arrows indicating the transcription directions. **C**, **D** ROC curves showing the performances of two-class models which distinguish HCC (**C**) or PDAC (**D**) from control samples. The results from 51 iterations for (**C**) and 53 iterations for (**D**) of leave-one-out cross-validation analysis were shown. **E** Bar plot showing the overall accuracy of three-class models which distinguish HCC, PDAC, and control samples. The results from 74 iterations of leave-one-out cross-validation analysis are shown. Methylation, methylation ratio of promoter and enhancer regions; Occupancy, nucleosome occupancy of 1 kb regions surrounding TSSs and polyadenylation sites; WPS, WPS of 1 kb regions surrounding TSSs and polyadenylation sites; Multimodal, the combination of all three types of features

As MESA took advantage of the non-disruptive nature of EM-seq to capture multimodal epigenetic information from a single assay, the multimodal approach was predicted to effectively perform on any cfDNA methylation sequencing assay of a similar nature. We tested this hypothesis on another bisulfite-free cfDNA sequencing method, cfDNA TAPS [27], which was applied to a cohort including 21 hepatocellular carcinoma (HCC) patients, 23 pancreatic ductal adenocarcinoma (PDAC) patients, and 30 non-cancer controls. As shown by a well-studied nucleosome array, the occupancy reported by DANPOS2 for cfDNA TAPS data was consistent with nucleosome profiles from lymphoblastoid cells (Fig. 6A), indicating cfDNA TAPS could capture nucleosome information as targeted EM-seq did. Despite the low sequence depth (mean coverage of 11.6 ×), we still observed occupancy changes between cancer and control samples for regions surrounding either TSSs or polyadenylation sites (Fig. 6B). Then, we extracted three types of features, including DNA methylation, nucleosome occupancy, and WPS. Next, we applied the same model training method for cohort 1 and cohort 2 to the cohort of cfDNA TAPS data (HCC vs. control; PDAC vs. control). Here, we did not include nucleosome fuzziness because it was inaccurate to calculate the fuzziness score when the sequencing depth was low. In line with the above results, we found that the multimodal model has the highest AUC compared with three single-modality models (Fig. 6C and D; AUC = 0.8683 for the HCC cohort and AUC = 0.8087 for the PDAC cohort). Since there were two cancer types in this dataset, we also trained three-class models to distinguish HCC, PDAC, and controls to demonstrate the outperformance of the multimodal model. We found that the multimodal model achieved an overall accuracy of 0.6892 (Fig. 6D), outperforming the three single-modality models. Moreover, the multimodal model had an overall high accuracy in distinguishing the two cancer types as shown by the confusion matrixes (Additional file 2: Fig. S6). Together, these results suggest that MESA’s integrated analysis of multimodal epigenetic features is widely applicable across multiple non-disruptive methylation sequencing protocols.

## Discussion

In this study, we present a comprehensive epigenetic analysis of cfDNA, aiming at improving the non-invasive early detection of human cancers. Our integrated model shows enhanced detection accuracy for colorectal, liver, and pancreatic cancers compared to single modality models in four cohorts with either EM-seq or cfDNA TAPS. Besides the good performance, another significant advantage of MESA is its flexibility. Although the biomarkers and model used in MESA are specific to a particular cancer type, the inclusion or exclusion of modalities is adaptable based on their performance when applied to different cancer types. For example, cancer types with relatively unchanged nucleosome occupancy may benefit only from integrating the remaining modalities. Removal of nucleosome occupancy features, in this case, could prevent confounding and unnecessary complexity. Therefore, this multimodal approach allows for the developing of an unbiased combinatorial prediction model. Furthermore, all four modalities are simultaneously captured in a single assay, offering full flexibility without the need to perform multiplex assays while minimizing potential batch effects and other technical biases in multiplex and separated assays.

MESA can also be applied to other clinical scenarios besides the basic classification question in this study. It can be used for a multi-cancer early detection test or test for high-risk individuals if trained on data of multiple cancer types or high-risk individuals. Furthermore, to utilize MESA for detecting minimal residual disease (MRD), we can train the models on patient samples with post-treatment recurrence status information, which can indicate MRD. In cases of early detection, where the proportion of healthy samples far exceeds the number of cancer samples in the training cohort, we may need to take additional steps during preprocessing to address the imbalance. This can be done using techniques such as the Synthetic Minority Over-Sampling Technique to generate synthetic samples by randomly sampling attributes from instances in the minority class before training the models. Additionally, performance metrics such as precision, recall, and F1 score can be used to evaluate the performance of classifiers in imbalanced datasets. A penalized classifier can also be used to give more weight to the minority class. These modifications can be easily applied to MESA by adjusting its parameters. In the case of small training cohorts, overfitting can become a major issue. To mitigate this issue, a regularization parameter can be added to the classifier, and its parameters can be fine-tuned on the training set to reduce overfitting. The optimal parameters can then be used to train a final model for clinical use.

A potential concern of this multimodal approach is that modalities might be highly correlated, thus not necessarily reflecting complementary information. In this paper, we showed that the predicted probabilities of individual modalities are not highly correlated. For example, although the nucleosome organization is related to WPS [22], nucleosomes can provide additional information. For example, nucleosome fuzziness can capture the cell heterogeneity at the chromatin level. Even if two samples have the same WPS profile, these samples may possess dramatically different nucleosome fuzziness in most regions. Therefore, they can still provide complementary information for the prediction model. We further note that, to our knowledge, this study introduces the measurement of nucleosome fuzziness and polyadenylation regions for the first time in cfDNA sequencing data analysis. Our results show that they both contribute to a better performance of the cancer detection model.

One limitation of our study is its relatively small sample size. Follow-up studies will be needed to strengthen the application of MESA in a wide variety of human cancers. However, despite the limitations, our study demonstrates a salient example of how targeted EM-seq of cfDNA captures multimodal epigenetic information and enables accurate cancer detection at a low relative cost. Our design provides a clinically practical method for liquid biopsy, especially for cancer types with few or no genetic changes. Moreover, for cohort 1, we observed better performances of the multimodal model for early-stage (I and II) than for late-stage (III and IV) patients (Additional file 1: Table S14). Although this observation may be biased by the relatively small sample size of each stage, it shows the possible advantages of MESA on early cancer detection. As cfDNA methylation-based liquid biopsies garner more attention and clinical use, MESA represents a widely applicable platform for improving non-invasive cancer detection.

## Conclusions

The multimodal epigenetic sequencing analysis (MESA), which integrates multiple epigenetic modalities, has demonstrated superior detection accuracy for colorectal, liver, and pancreatic cancers compared to models based on a single modality. This enhanced detection has been validated in four distinct cohorts using either EM-seq or cfDNA TAPS techniques. As a result, MESA represents a major advancement in non-invasive cancer detection by leveraging comprehensive and complementary epigenetic profiles of cfDNA.

### Supplementary Information

**Additional file 1:** **Table S1.** Summary of studies based on non-destructive cfDNA methylation sequencing. **Table S2.** Cilinical information and sequencing statistics for Cohort 1. **Table S3.** Cilinical information and sequencing statistics for Cohort 2. **Table S4.** Cilinical information and sequencing statistics for Cohort 3. **Table S5.** The whole targeted panel for Cohort 1. **Table S6.** The nucleosome organization targeted panel for Cohort 1. **Table S7.** The whole targeted panel for Cohort 2. **Table S8.** The nucleosome organization targeted panel for Cohort 2. **Table S9.** The whole targeted panel for Cohort 3. **Table S10.** The nucleosome organization targeted panel for Cohort 3. **Table S11.** Summary of nucleosome-associated features in cfDNA-based cancer liquid biopsy literature. **Table S12.** Sensitivity and F1 score of models based on different modalities. **Table S13.** Confusion matrix of models based on different modalities. **Table S14.** The AUC values of ROC curves for different models and cancer patients in different stages.

**Additional file 2:** **Fig. S1.** Scatter plots showing PC1 and PC2 from PCA of the methylation ratio of all target CpG sites of Cohort 1 (A) and Cohort 3 (B). Different sample collecting sites are corlored by different colors. **Fig. S2.** Fragment length distribution of sequenced cfDNA fragments for Cohort 2 (A) and Cohort 3 (B). A peak value (black dashed line) at 169 bp or 166 bp is consistent with the association with nucleosome. Results in this figure are based on merged targeted EM-seq data of all healthy controls from Cohort 2 and Cohort 3 respectively. **Fig. S3.** The distribution of dinucleotide fraction across 147 bp fragments and the flanking genomic regions for Cohort 2 (A) and Cohort 3 (B). Results in this figure are based on merged targeted EM-seq data of all healthy controls from Cohort 2 and Cohort 3 respectively. **Fig. S4.** Average SMAC-seq profile around all human PA sites collected in PolyA_DB (version 3). **Fig. S5.** Comparisons between nucleosome fuzziness and WPS/OCF. (A-B) Histograms showing the distribution of per sample Spearman correlations between nucleosome fuzziness and WPS (A) or OCF (B). (C) Heatmap showing the predicted probabilities of models based on the three modalities for each sample. The probability represents the predicted probability of classifying the sample to the cancer group. All these analyses were done in Cohort 1. **Fig. S6.** Confusion matrices for three-class models based on different modalities for cfDNA TAPS dataset. Methylation, methylation ratio of promoter and enhancer regions; Occupancy, nucleosome occupancy of 1 kb regions surrounding TSSs and PASs; WPS, WPS of 1 kb regions surrounding TSSs and PASs; Multimodal, the combination of all three types of features.

## Data Availability

All processed data used to generate the results are available at Zenodo [65]. The raw sequencing reads are available from the European Genome-phenome Archive (EGA) through accession number EGAS00001006462 (https://ega-archive.org/studies/EGAS00001006462) [66] and EGAS50000000052 (https://ega-archive.org/studies/EGAS50000000052) [67]. MESA source code is available at GitHub via URL https://github.com/ChaorongC/MESA[68]. All the codes and data used to reproduce all the major results in this manuscript is available from https://rpubs.com/LiYumei/926228[69].
