# Supplementary material for: Multimodal epigenetic sequencing analysis (MESA) of cell-free DNA for non-invasive colorectal cancer detection
Source: Genome Med. 2024 Jan 16;16:9. doi: 10.1186/s13073-023-01280-6 (PMC10790422; doi:10.1186/s13073-023-01280-6)
Supplement: Supplementary file 2 — Additional file 2: Fig. S1. Scatter plots showing PC1 and PC2 from PCA of the methylation ratio of all target CpG sites of Cohort 1 (A) and Cohort 3 (B). Different sample collecting sites are corlored by different colors. Fig. S2. Fragment length distribution of sequenced cfDNA fragments for Cohort 2 (A) and Cohort 3 (B). A peak value (black dashed line) at 169 bp or 166 bp is consistent with the association with nucleosome. Results in this figure are based on merged targeted EM-seq data of all healthy controls from Cohort 2 and Cohort 3 respectively. Fig. S3. The distribution of dinucleotide fraction across 147 bp fragments and the flanking genomic regions for Cohort 2 (A) and Cohort 3 (B). Results in this figure are based on merged targeted EM-seq data of all healthy controls from Cohort 2 and Cohort 3 respectively. Fig. S4. Average SMAC-seq profile around all human PA sites collected in PolyA_DB (version 3). Fig. S5. Comparisons between nucleosome fuzziness and WPS/OCF. (A-B) Histograms showing the distribution of per sample Spearman correlations between nucleosome fuzziness and WPS (A) or OCF (B). (C) Heatmap showing the predicted probabilities of models based on the three modalities for each sample. The probability represents the predicted probability of classifying the sample to the cancer group. All these analyses were done in Cohort 1. Fig. S6. Confusion matrices for three-class models based on different modalities for cfDNA TAPS dataset. Methylation, methylation ratio of promoter and enhancer regions; Occupancy, nucleosome occupancy of 1 kb regions surrounding TSSs and PASs; WPS, WPS of 1 kb regions surrounding TSSs and PASs; Multimodal, the combination of all three types of features. [file 13073_2023_1280_MOESM2_ESM.pdf]

Fig. S1

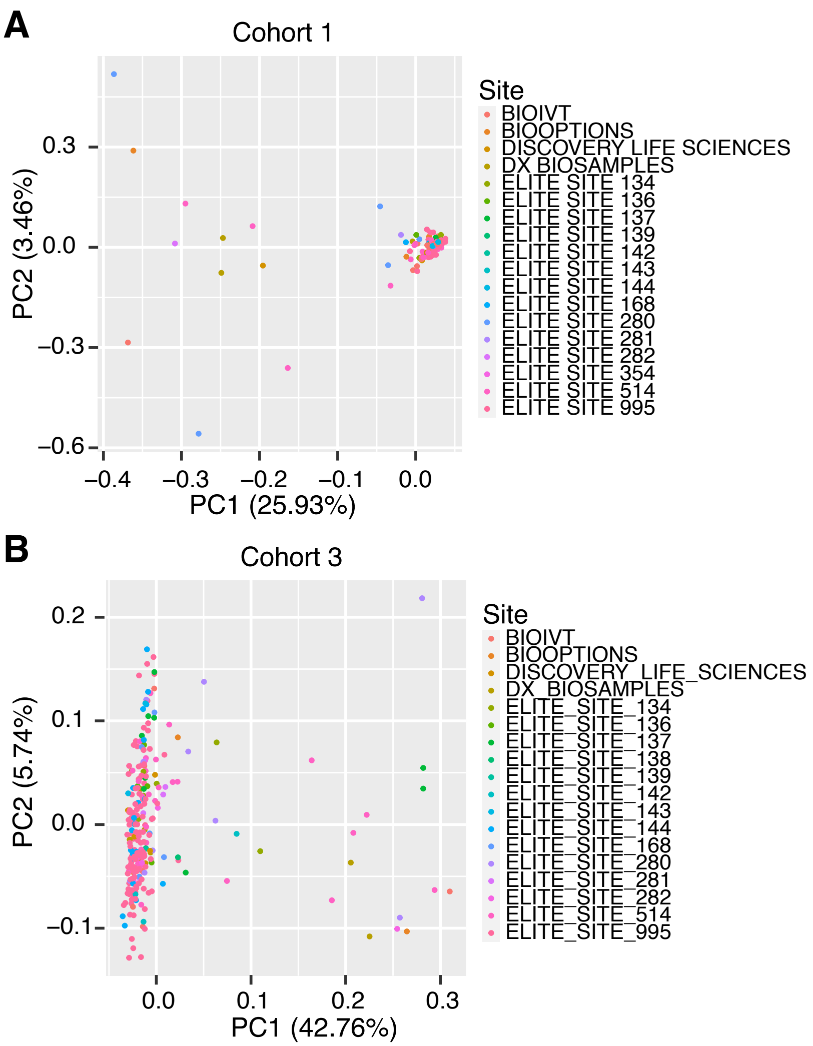

Fig. S1. Scatter plots showing PC1 and PC2 from PCA of the methylation ratio of all target CpG sites of Cohort 1 (A) and Cohort 3 (B). Different sample collecting sites are corlored by different colors.

Fig. S2

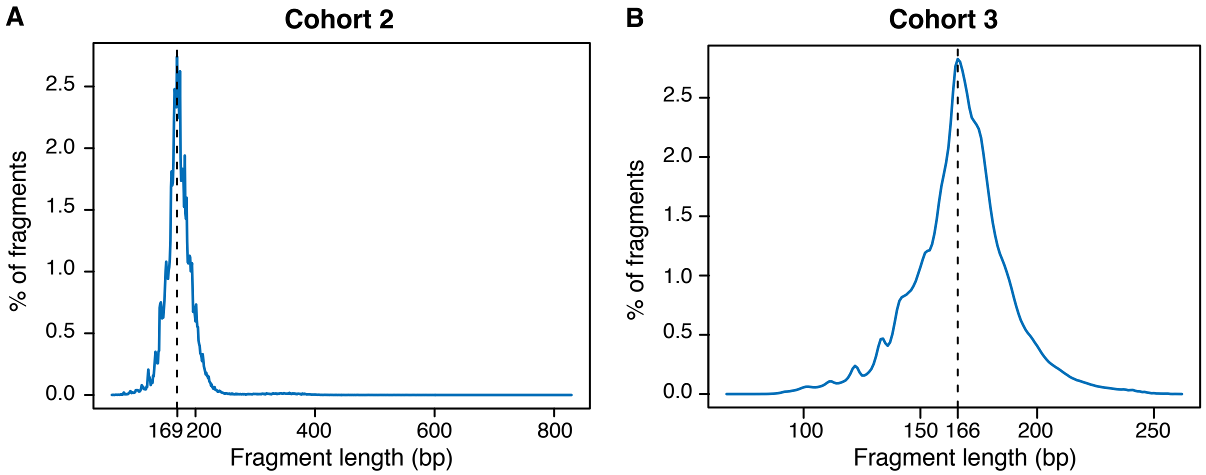

Fig. S2. Fragment length distribution of sequenced cfDNA fragments for Cohort 2 (A) and Cohort 3 (B). A peak value (black dashed line) at 169 bp or 166 bp is consistent with the association with nucleosome. Results in this figure are based on merged targeted EM-seq data of all healthy controls from Cohort 2 and Cohort 3 respectively.

Fig. S3

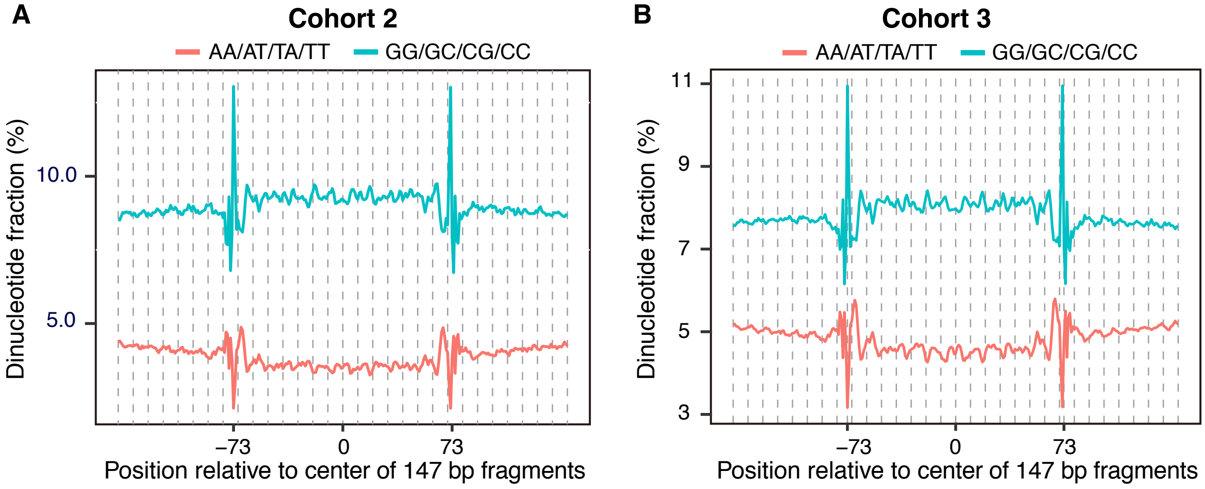

Fig. S3. The distribution of dinucleotide fraction across 147 bp fragments and the flanking genomic regions for Cohort 2 (A) and Cohort 3 (B). Results in this figure are based on merged targeted EM-seq data of all healthy controls from Cohort 2 and Cohort 3 respectively.

Fig. S4

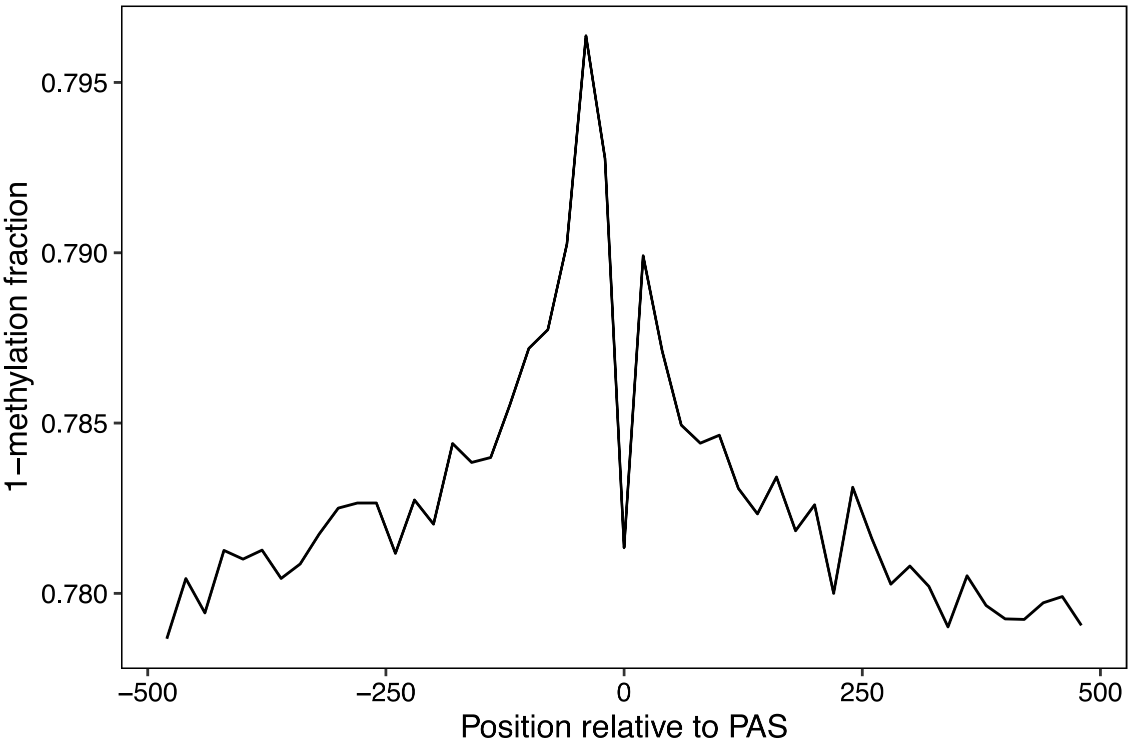

Fig. S4. Average SMAC-seq profile around all human PA sites collected in PolyA_DB (version 3).

Fig. S5

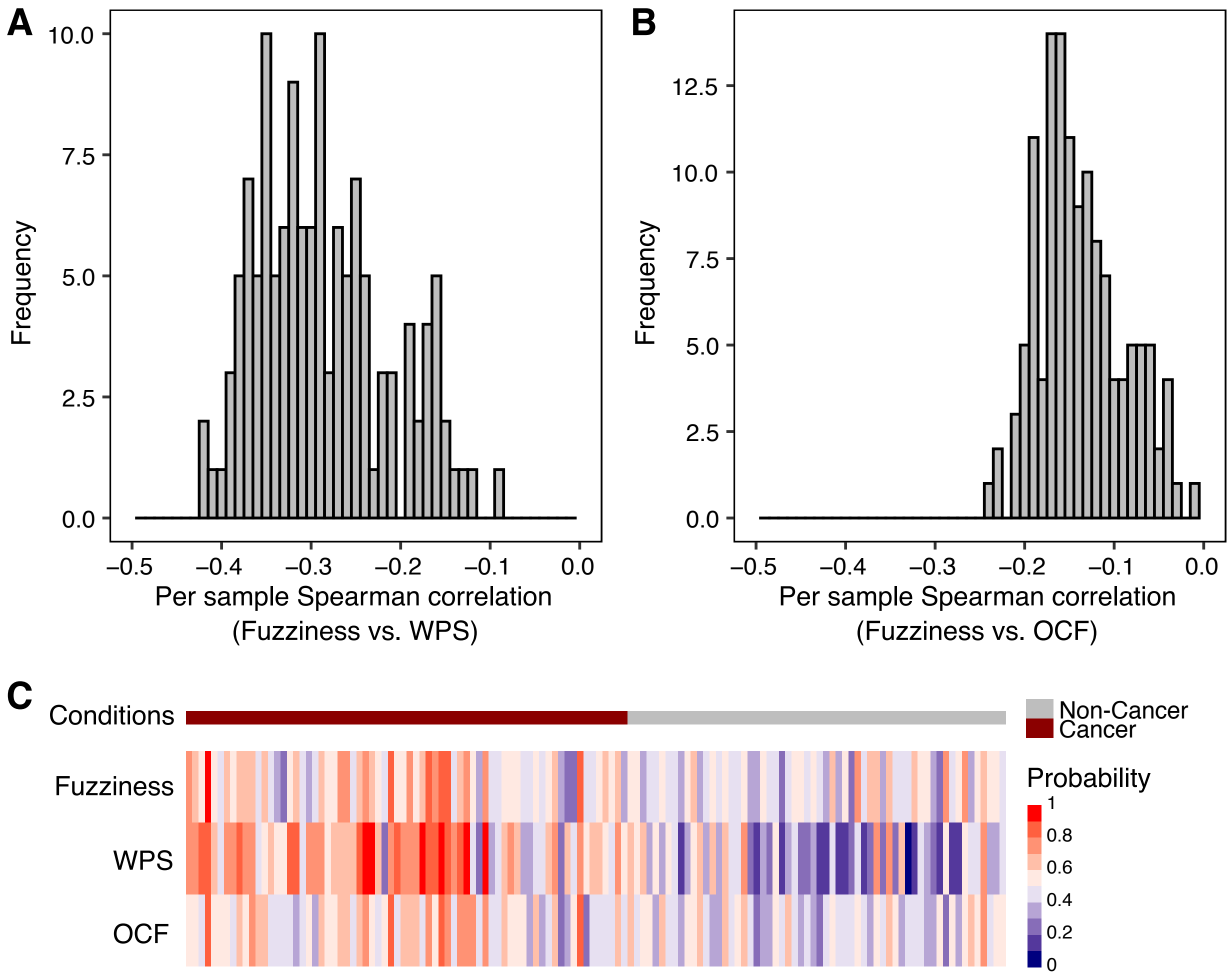

Fig. S5. Comparisons between nucleosome fuzziness and WPS/OCF. (A-B) Histograms showing the distribution of per sample Spearman correlations between nucleosome fuzziness and WPS **(A)** or OCF **(B)**. **(C)** Heatmap showing the predicted probabilities of models based on the three modalities for each sample. The probability represents the predicted probability of classifying the sample to the cancer group. All these analyses were done in Cohort 1.

Fig. S6

A **Multimodal**

		Predicted		
		Control	HCC	PDAC
Actual	Control	24	2	4
	HCC	2	14	5
	PDAC	7	3	13

B **Methylation**

		Predicted		
		Control	HCC	PDAC
Actual	Control	23	1	6
	HCC	6	12	3
	PDAC	6	4	13

C **Nucleosome occupancy**

		Predicted		
		Control	HCC	PDAC
Actual	Control	24	1	5
	HCC	3	14	4
	PDAC	12	4	7

D **WPS**

		Predicted		
		Control	HCC	PDAC
Actual	Control	17	5	8
	HCC	8	10	3
	PDAC	8	6	9

Fig. S6. Confusion matrices for three-class models based on different modalities for cfTAPS dataset. Methylation, methylation ratio of promoter and enhancer regions; Occupancy, nucleosome occupancy of 1 kb regions surrounding TSSs and PASs; WPS, WPS of 1 kb regions surrounding TSSs and PASs; Multimodal, the combination of all three types of features.
